# Epithelial Antimicrobial Peptides: Guardian of the Oral Cavity

**DOI:** 10.1155/2014/370297

**Published:** 2014-11-11

**Authors:** Mayank Hans, Veenu Madaan Hans

**Affiliations:** ^1^Department of Dentistry, ESIC Medical College and Hospital, Faridabad, Haryana 121001, India; ^2^Department of Periodontics, Faculty of Dental Sciences, SGT University, Gurgaon, Haryana 122505, India

## Abstract

Gingival epithelium provides first line of defence from the microorganisms present in dental plaque. It not only provides a mechanical barrier but also has an active immune function too. Gingival epithelial cells participate in innate immunity by producing a range of antimicrobial peptides to protect the host against oral pathogens. These epithelial antimicrobial peptides (EAPs) include the *β*-defensin family, cathelicidin (LL-37), calprotectin, and adrenomedullin. While some are constitutively expressed in gingival epithelial cells, others are induced upon exposure to microbial insults. It is likely that these EAPs have a role in determining the initiation and progression of oral diseases. EAPs are broad spectrum antimicrobials with a different but overlapping range of activity. Apart from antimicrobial activity, they participate in several other crucial roles in host tissues. Some of these, for instance, *β*-defensins, are chemotactic to immune cells. Others, such as calprotectin are important for wound healing and cell proliferation. Adrenomedullin, a multifunctional peptide, has its biological action in a wide range of tissues. Not only is it a potent vasodilator but also it has several endocrine effects. Knowing in detail the various bioactions of these EAPs may provide us with useful information regarding their utility as therapeutic agents.

## 1. Introduction

Oral cavity is a diverse ecosystem where several microbes, commensal and pathogenic, interact and proliferate in the moist and warm environment. It is perhaps the only ecosystem of the human body which is dynamic, open, and close at the same time. The uniqueness of the oral cavity also lies in the presence of specialized interface which in the hard tissue (tooth) extrudes through the underlying soft tissue (gingival epithelium) [1]. The gingival epithelium constitutes oral epithelium, sulcular epithelium, and junctional epithelium. Since the tooth surface is invariably covered by a layer of microbial biofilm termed “dental plaque,” the surrounding epithelium is continuously exposed to microorganisms and their products [2]. The role of gingival epithelium has been under the scanner because of this close interaction. Previously considered to be an innocent bystander, its active role in host microbial interaction has been elucidated with recent research [3].

Gingival epithelium works in many ways for the protection of underlying connective tissue. First, it provides a physical barrier which does not allow microbes to invade while permitting selective transaction with oral environment. Second, upon interaction with microbes it secretes cytokines as well as chemokines to activate the influx of neutrophils and other immune cells into the sulcular area [4]. Third, the gingival epithelium has been found to be a source of antimicrobial products termed epithelial antimicrobial peptides (EAP). These EAPs are considered to be important for the innate immunity of the host. These peptides are known to possess a wide spectrum of antimicrobial activity acting against Gram-positive and Gram-negative bacterial species as well as yeast and some viruses [5]. Thereby, EAPs have the potential to prevent various oral diseases of microbial origin such as periodontitis, dental caries, candidiasis, and herpetic gingivostomatitis.

Since gingival epithelial cells are in close proximity with microbes and their virulence factors, the response of epithelium to these insults determines the health of the gingival sulcus. Several families of antimicrobial peptides have been identified in oral cavity which includes *α*-defensins, *β*-defensins, calprotectin, adrenomedullin, histatins, and cathelicidin. Of these *α*-defensins are of nonepithelial origin, with their major source being the neutrophils migrating into the gingival sulcus as primary host response, whereas histatins are secreted in saliva, produced by parotid and submandibular salivary gland duct cells [6, 7]. The EAPs work in tandem with other salivary antimicrobial agents such as histatins, immunoglobulins, and lysozyme to strengthen first line of host defence. These antimicrobial peptides are expressed in oral epithelium constitutively and are inducible; thus, they have both functions, of surveillance and of host defence [8].

## 2. Epithelial Antimicrobial Peptides in Oral Cavity

Several antimicrobial peptides produced by epithelial cells are widespread in animal kingdom ranging from Cecropins in insects to Magainins in frogs and Bactenecins in cattle [9–11]. All these antimicrobial peptides although different in structure and amino acid composition share a common trait of being broad spectrum antibiotics and essentially the first line of defence for host. First mammalian antimicrobial peptide identified in oral epithelium was the lingual antimicrobial peptide (LAP) by the works of Schonwetter et al. on bovine tongue [12]. LAP was found to have antibiotic activity against Gram-positive and Gram-negative bacteria as well as antifungal properties [12]. After LAP, several more EAPs have been identified in mammals including humans. The EAPs identified in humans are *β*-defensin family, cathelicidin (LL-37), calprotectin, and adrenomedullin. The major characteristics of these EAPs are compared in Table 1.

## 3. The ***β***-Defensin Family

Beta-defensins are universally expressed in all human epithelial cells [13]. In the oral cavity, they have been found in oral mucosa, gingiva, and tongue epithelium along with salivary glands [14]. They were first described in bovine tracheal pseudostratified epithelium [15]. Beta-defensins share certain structural similarities with *α*-defensins although they are a bit larger than *α*-defensins. However, both *α*- and *β*-defensins are cationic in nature because of presence of arginine and lysine residue in their structure [16]. Out of several *β*-defensins (hBDs) known, four have been characterized elaborately and termed as hBD-1, hBD-2, hBD-3, and hBD-4. Out of this only hBD-1, hBD-2, and hBD-3 are expressed in the oral cavity [17]. hBD-1 and hBD-2, found in the suprabasal layer of epithelium, can be localized to differentiated epithelial cells, whereas hBD-3 is expressed in undifferentiated epithelial cells found in the basal layer of epithelium [18, 19]. Apart from hBD-1, which is constitutively expressed, the rest of the *β*-defensins are inducible upon stimulation with microbes or proinflammatory cytokines [20]. Recently another *β*-defensin hBD-9 has been identified and localized in gingival epithelium [21].

### 3.1. Genes Encoding *β*-Defensin

The first human *β*-defensin was isolated from hemofiltrate passing through kidney [22]. The gene encoding for hBD-1 is present on chromosome 8 and is termed DEFB1. It lies in close proximity to the neutrophil *α*-defensin gene (DEFA 1), arranged approximately 100–150 kilobases apart. DEFB1 has two exons and one intron which is translated into a hBD-1 propeptide [23]. This propeptide undergoes posttranslational modifications and forms several hBD-1 mature peptides upon cleavage which are about 36–47 amino acids long [24].

The second human *β*-defensin was first isolated in the psoriatic skin keratinocytes [25]. hBD-2 is encoded via gene EFB4 which is also located on chromosome 8 in close proximity to DEFB1. Similar to DEFB1, it has two exons and one intron which encodes a signal peptide which is 23 amino acids long and a mature peptide which is 41 amino acids long [26].

DEFB103, which is the gene for hBD-3, has been localized on chromosome 8 above the hBD-2 gene. The translational product of this gene consists of a propeptide with a signal peptide domain and a mature peptide of 22 and 45 amino acids in length, respectively. The amino acid sequence of hBD-3 shares some amount of similarity with hBD-2 [27].

### 3.2. Induction of Genetic Expression

Human *β*-defensin-1 is expressed at a low level within gingival and buccal epithelium, dental pulp, and salivary gland tissue [5]. It has been observed that hBD-1 is not regulated in response to infection or other stimuli, which means that it is not inducible. Otherwise expressed at a very low level, hBD-2 and hBD-3 are, on the other hand, inducible in response to microbes and their products [28]. Their amplified expression in response to infection has already been confirmed in gingivitis and periodontitis [29, 30]. IL-1*β*, tumor necrosis factor (TNF)-*α*, and IL-17 have been implicated in the induction of hBD-2 and hBD-3 expression in epithelial cells [31]. Their induction via cytokines confirms their role in innate immunity. IL-17 also induces *β*-defensin expression against the colonization of* Candida* in oral cavity [32]. Thus, *β*-defensins expressed within the oral cavity not only check an overgrowth of commensal microorganisms but also prevent colonization of pathogens.

### 3.3. Structure

The structure of *β*-defensins is characterized by an antiparallel *β*-pleated sheet stabilization via three intramolecular disulphide bonds formed between six cysteine amino acids. Also, hBD-2 and hBD-3 contain an *α*-helical domain at their N terminus. Since *β*-defensin peptide contains both hydrophilic and hydrophobic domains in their structure, they are amphiphatic in nature [33]. The amino acid sequences of these defensins and other oral EAPs are provided in Table 2.

### 3.4. Antimicrobial Activity

The salivary concentration of hBD-1 and hBD-2 varies in a range of nondetectable to 39 ng/mL and 33 ng/mL, respectively [34]. On the other hand, hBD-3 exists at a concentration range of 0.31 *μ*g/mL in saliva [35]. Several in vitro assays have revealed *β*-defensins to be active against broad range of microbes including Gram-positive and Gram-negative bacteria, enveloped viruses, and fungi [36–39]. They are also efficacious against periodontal pathogens such as* Aggregatibacter actinomycetemcomitans* and* Fusobacterium nucleatum* [37]. A recent study demonstrated extermination of* F. nucleatum* with in gingival epithelial cells via hBD-2 and hBD-3 [40]. Their spectrum of activity also includes* Candida albicans* along with other* Candida* spp. in oral cavity [38]. In an experiment carried out to assess the antimicrobial activity of hBD-1, hBD-2, and hBD-3 against periodontopathic and cariogenic bacteria, it was suggested that Gram-negative bacteria except* F. nucleatum* were less susceptible to antimicrobial peptides than Gram-positive organisms. Except for hBD-1, all peptides demonstrated 100% bactericidal activity with concentration of >10 mg/L of peptides. hBD-1 and hBD-2 were significantly less effective than hBD-3 in their antimicrobial activity. The minimum inhibitory concentration (MIC) for hBD-3 was around 100–200 mg/L for* A. actinomycetemcomitans*,* P. gingivalis*, and* Prevotella intermedia*, whereas it was 12.5 mg/L for* F. nucleatum* [41]. In another study, the MIC of hBD-2 and hBD-3 was found to be in a range of 3.9 to >250 *μ*g/mL and 1.4 to >250 *μ*g/mL, respectively, against* A. actinomycetemcomitans*,* F. nucleatum*,* P. gingivalis*,* Peptostreptococcus micros*,* Streptococcus mutans*,* S. sanguis*, and* Candida* spp. [36].

It has also been observed that certain periodontal pathogens have developed resistance against *β*-defensins, thereby enhancing their pathogenicity. These pathogens include* P. gingivalis* and* Treponema denticola* [42, 43]. It is assumed that* T. denticola* interacts with the signal transduction pathway to suppress the expression of *β*-defensins [43]. Also, recent data demonstrates that hBD-2 and hBD-3 expression in adult gingival epithelial cells inactivates human immunodeficiency virus (HIV) suggesting an important role of *β*-defensins in antiviral defence [39].

### 3.5. Mechanism of Antimicrobial Action

**Figure 1:**
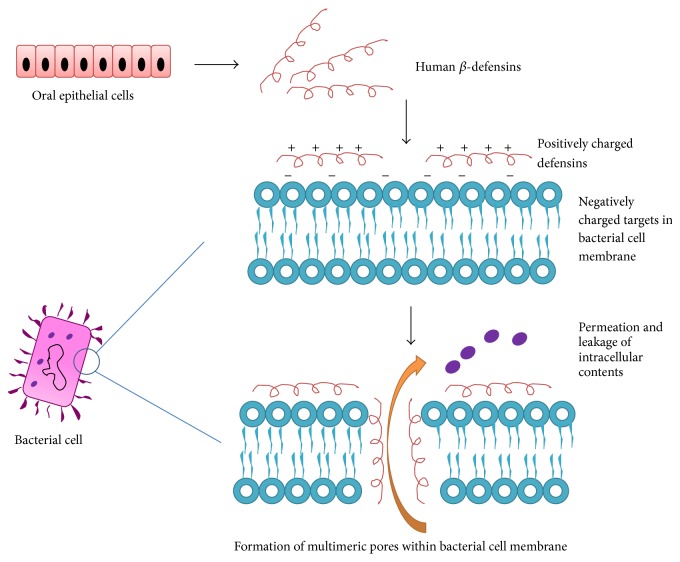
Mechanism of antimicrobial action of human beta-defensins.

*β*-defensins belong to a group of cationic antimicrobial peptides (CAPs), known to target the bacterial cell membrane. Since *β*-defensins are positively charged, they bind to the negatively charged sites on the bacterial cell membrane. In Gram-negative bacteria, the target is lipopolysaccharide (LPS), whereas in Gram-positive bacteria it is teichoic acid. Apart from these, membrane rich phospholipids (phosphatidyl glycerol) which are common to both Gram-positive and Gram-negative bacteria are also targeted by *β*-defensins. As the eukaryotic cell membrane is rich in phosphatidylcholine rich phospholipids which are zwitterionic in nature, they are exempted from the action of *β*-defensins [8]. It has been hypothesized that defensins upon interaction with anion lipids of bacterial cell membrane leads to formation of multimeric pores and permeation of the membrane (Figure 1). This permeation leads to the loss of vital contents of the bacterial cell leading to cell death [44].

### 3.6. Other Roles

**Figure 2:**
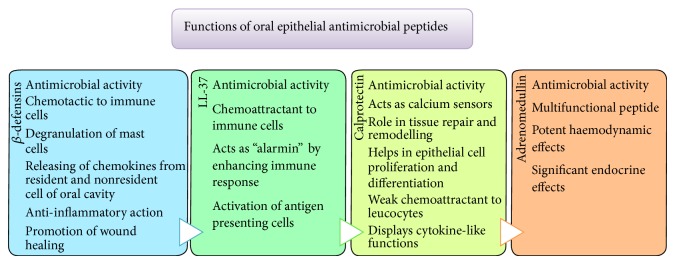
Functions of oral epithelial antimicrobial peptides.

Various roles of *β*-defensins are summarized in Figure 2. Since *β*-defensins exhibit similarities to cytokines structurally, they show potent chemotactic activity to a range of immune cells [45, 46]. This activity takes place at a much lower level of *β*-defensin concentration than required for its antimicrobial activity. hBD-1 activates immature dendritic cells and memory T cells, hBD-2 is able to recruit mast cells and neutrophils, and hBD-3 is chemotactic for neutrophils, dendritic cells, mast cells, and monocytes. hBD-2 and hBD-3 also induce degranulation of mast cells [47–49]. Recently, it has been demonstrated that *β*-defensins are able to bind to CXCR 4 receptors on T cells, leading to internalization of receptor. This receptor is crucial for HIV-1 infection of T cell, thereby its internalization provides a defence against HIV-1 infection [39].

Beta-defensins have a role in inducing the resident as well as nonresident cells to release chemokines. They induce the release of several chemokines from keratinocytes such as monocyte chemotactic protein-1, interferon-*γ* inducible protein-10, and macrophage inflammatory protein 3*α* [50]. Beta-defensins are known to inhibit production of chemokines and cytokines such as TNF-*α* and IL-6, thereby exhibiting a role in anti-inflammatory activities and suppression of immune response [51, 52]. They also have a role in promotion of wound healing, where the growth factors secreted by various immune cells in the area of injury can stimulate keratinocytes to release hBD-3. These growth factors include insulin-like growth factor- (IGF-) 1, transforming growth factor- (TGF-) *α*, and epidermal growth factor (EGF) [53]. Also, *β*-defensins act as a connecting link between innate and adaptive immunity. Epithelial cells release *β*-defensins along with chemokines and cytokines, to signal Langerhans cells which are antigen presenting cells within the epithelium. Also, hBD-1 and hBD-2 act as a chemoattractant for T cells and dendritic cells [54].

## 4. Human Cathelicidin (LL-37)

Cathelicidin family of antimicrobial peptides consists of a “cathelin” domain at their N terminus and a mature peptide at their C terminus [55]. The amino acid sequence of the cathelin domain is highly conserved and thereby similarity is observed despite species or cells it is obtained from [56]. The mature peptide however demonstrates considerable variation in its size, amino acid sequence, and three-dimensional structures [57]. The cathelin domain derives its name from a porcine neutrophil protein of the same name, since both share sequence homology [57]. The only antimicrobial peptide of Cathelicidin family expressed in humans is LL-37. It was cloned from human bone marrow cDNA and derives its name from its length of 37 amino acids with two leucine residues in the beginning (Table 2) [58]. It is a cationic antimicrobial peptide of 18 Kda size, therefore also termed hCAP 18 [59]. It is expressed in epithelial cells lining the respiratory, gastrointestinal, and urogenital tract as well as oral cavity, although its main source in oral cavity is from neutrophilic granules and to a lesser extent from epithelial cells [18]. In oral cavity, LL-37 is expressed in inflamed gingival tissues, buccal mucosa, and tongue epithelium [60]. It has also been identified in saliva and GCF [61, 62]. The concentration of LL-37 is found to increase with increasing depth of gingival sulcus [63]. It has also been proposed that LL-37 detected in gingival epithelium may be the product of neutrophil migration through gingival epithelium rather than epithelial cells themselves [18].

### 4.1. Gene Encoding LL-37

The gene encoding LL-37 is located on chromosome 3 at location 3p21.3. It has been named cathelicidin antimicrobial peptide (CAMP) gene. LL-37 gene has four exons and three introns. First three exons encode for the signal sequence and cathelin region of the peptide while the fourth exon translates into mature peptide [54]. In intron and promoter region of LL-37, there exist binding sites for acute phase response factors, which establish the upregulation of LL-37 in inflammation [64].

### 4.2. Induction of Gene Expression

LL-37 expression in various cell types has been found to be upregulated on exposure to growth factors, differentiating agents, and microorganisms. Insulin-like growth factor-1 which is known to promote wound healing upregulates LL-37 expression [65]. Also, vitamin D which is a differentiating agent has been found to amplify LL-37 activity [66]. Increased level of LL-37 in gingival tissues in response to inflammation correlates positively with depth of gingival crevice [63]. In a comparative study, levels of LL-37 in GCF were found to be significantly elevated in chronic periodontitis patients than in gingivitis patients and healthy volunteers [67].

### 4.3. Structure

The cathelicidin gene in humans is translated into an inactive precursor protein termed as hCAP-18. Upon posttranslational processing an active C terminus peptide with 37 amino acids is released from precursor protein. This cleavage is carried out via proteolytic enzyme elastase or proteinase-3 [68, 69]. This peptide has a net positive charge at physiologic pH and more than 50% of its residues are hydrophilic in nature. Structurally, it exists as a random coil in aqueous solutions. Many of its amino acids form intramolecular hydrogen bonds, acquiring an *α*-helix secondary structure. It is supposed that antibacterial activity of LL-37 is correlated with *α*-helicity [70].

### 4.4. Antimicrobial Activity

Found in saliva in a concentration of 0.14–3 *μ*g/mL, LL-37 is active against both Gram-negative and Gram-positive bacteria including established periopathogens* P. gingivalis* and* A. actinomycetemcomitans* [71]. Its MIC was 30–60 *μ*g/mL and 125 *μ*g/mL against* A. actinomycetemcomitans* and* P. gingivalis*, respectively [37]. LL-37 maintains its antimicrobial activity even in presence of gingipains with some salivary components providing it with protection [72]. It is ascertained that LL-37 inhibits the inflammatory response in gingival fibroblasts to* P. gingivalis* and its products [73]. LL-37 binds directly to bacterial lipopolysaccharide [59]. Morbus Kostmann syndrome is a genetic form of periodontal disease which was shown to have a near absence of LL-37 [74]. This suggests the importance of LL-37 as an antimicrobial peptide in oral cavity.

### 4.5. Mechanism of Antimicrobial Action

**Figure 3:**
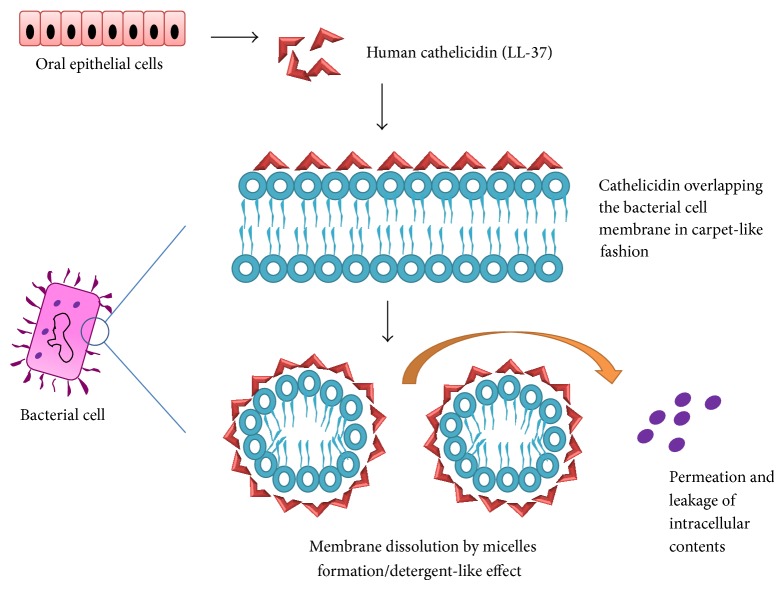
Mechanism of antimicrobial action of human cathelicidin (LL-37).

LL-37 belongs to the category of amphiphatic *α*-helical antimicrobial peptides. It has been proposed that these peptides do not function via formation of transmembrane pores within the lipid biolayers of microbial cell membrane as is the case with defensins. Although the exact mechanism of action of Cathelicidins is not clear, some members of this family overlap the bacterial cell membrane in a carpet-like manner and dissolve it similar to a detergent by micelles formation [75] (Figure 3).

### 4.6. Other Roles

LL-37 acts as a chemoattractant and causes influx of neutrophils, monocytes, and T cells to the site of inflammation [76]. Some researchers believe that it acts as an “alarmin” rather than an antimicrobial by enhancing the immune response leading to activation of antigen presenting cells [77]. The major roles of LL-37 are described in Figure 2.

## 5. Calprotectin

Calprotectin, also known as calgranulin, is a heterodimer of two anionic peptides MRP8 and MRP14 [78]. Several nomenclatures have been proposed for these individual peptides as well as calprotectin dimer but controversy regarding nomenclature still exists. These belong to S100 family of calcium binding proteins [79]. Calprotectin has been found to be constitutively expressed in cells of immune function such as neutrophils, monocytes, macrophages, and epithelial cells [80–82]. Its level increases in plasma, saliva, and synovial fluid during infectious and inflammatory diseases [83–85]. Also, it is highly responsive to any kind of stress in epidermal cells [86].

### 5.1. Gene Encoding for Calprotectin

The HGNC approved names of genes encoding for MRP8 and MRP14 are S100A8 and S100A9, respectively. The genes are from a closely located cluster of thirteen genes on chromosome 1 at location 1q21 [87]. The organization of these genes is evolutionarily conserved. Both of these peptides have three exons separated by two introns. Exon 1 is untranslated, whereas exons 2 and 3 encode into an N-terminal and a C-terminal EF hand motif, respectively [88].

### 5.2. Induction of Genetic Expression

Calprotectin expression is upregulated in epithelial cells upon induction of stress and exposure to ultraviolet radiation and in wound healing [86]. Also, its level heightens via exposure to complement factor C5a and proinflammatory cytokines such as TNF-*α*, IL-1*β*, and IL-6 [89]. Calprotectin levels have been found to positively correlate with severity of periodontitis in gingival crevicular fluid (GCF) [90]. The origin of calprotectin in GCF is from sulcular epithelium as well as immune cells. Calprotectin expression amplifies with increased pocket depth as well as with increasing levels of aspartate aminotransferase, IL-1*β*, prostaglandin E2, and collagenase in GCF [91]. Immunohistochemical analysis reveals its increased expression in pocket epithelium of periodontitis patients [92].

### 5.3. Structure

Calprotectin exists as a noncovalently complexed heterodimer of two calcium and zinc binding proteins MRP8 and MRP14. Calprotectin is a 36.5 KDa molecule with two subunits: 8.3 KDa light chain MRP8 and 13.3 KDa heavy chain MRP14. MRP8 is a 93-amino-acid-long chain whereas MRP14 has 114 amino acids, respectively (Table 2). Each monomer has two helix-loop-helix calcium ion (Ca^2+^) binding domains, EF hands and a hinge region separating two hydrophobic regions [93]. The C-terminal region contains a zinc ion (Zn^2+^) binding site as well as a phosphorylation site [94].

### 5.4. Antimicrobial Activity

Calprotectin is present in a concentration of 22 mg/L in stimulated whole saliva whereas its concentration in parotid saliva is around 3.2 mg/L [95]. The biostatic activity of calprotectin has been demonstrated in few bacteria such as* Staphylococcus aureus*,* S. epidermis*, and* Escherichia coli*. Against these organisms, calprotectin has a MIC of 64–256 mg/L. For* Cryptococcus neoformans*, the MIC was found to be 4–128 mg/L and 2–4 times this concentration was fungicidal [96]. It was observed in an* in vitro* experiment that calprotectin when expressed in oral epithelial cells confers protection against invasion by* P. gingivalis*, an established periodontal pathogen [97]. Its antifungal activity against* C. albicans* is also beneficial in maintaining oral health [98]. Similarly, increased calprotectin expression has been detected in oral keratinocytes infected with Epstein-Barr virus and herpes simplex virus [99].

### 5.5. Mechanism of Antimicrobial Action

The antimicrobial activity of calprotectin can be attributed to its trace metal binding activity. As discussed before, calprotectin has sites for binding of calcium and zinc ions as well as other dipositively charged trace metal ions such as copper and manganese [100]. Whenever epithelial cells are encountered with microbial insults, calprotectin is released into the interstitial milieu. There it may act via chelation of zinc or other divalent ions. These ions are essential for usual microbial functioning; thus, calprotectin provides a growth inhibitory type of host defence as an antimicrobial agent [101].

### 5.6. Other Roles

Calprotectin is termed as calcium sensor together with other S100 proteins as they change their conformation in response to calcium influx [102]. It appears to have a role in tissue repair and remodeling. It has been hypothesized that calprotectin participates in epithelial cell proliferation as well as differentiation [103]. It acts as a chemoattractant for leucocytes although being weak [104]. They display cytokine-like functions and act as ligand for toll-like receptor- (TLR-) 4 and receptor for advanced glycated end products (RAGE) [105]. Figure 2 summarizes various roles of calprotectin.

## 6. Adrenomedullin

Adrenomedullin belongs to the category of regulatory peptides with a wide array of biological action. Adrenomedullin was first purified and sequenced in 1993 from many peptides extracted from pheochromocytoma of a Japanese patient. It was termed adrenomedullin since it was derived from adrenal medulla [106]. But now it is known that it is a ubiquitous peptide with many cell types producing the peptide. Since the time of its discovery, adrenomedullin has been measured in various diseases such as cardiovascular, liver and renal diseases, and preeclampsia [107, 108]. It is believed that rise in levels of adrenomedullin is actually a consequence rather than cause of pathology.

### 6.1. Gene Encoding Adrenomedullin

Adrenomedullin is expressed in many cells including adrenal medulla, kidney, and lung as well as epithelial lining of skin, gut, and oral cavity [109, 110]. It is produced as a precursor molecule preproadrenomedullin. The gene encoding for preproadrenomedullin is termed adrenomedullin (ADM) gene which is located on chromosome 11. This gene is composed of four exons and three introns. There are binding sites for NF-*κ* *β* on promoter region of this gene [111]. Preproadrenomedullin is 185 amino acids long, with a 21-amino-acid-long N terminal signal peptide and a 20-amino-acid-long amidated peptide [106]. It has also been hypothesized that another biologically active peptide adrenotensin may also be product of adrenomedullin gene. Adrenotensin is proteolytic product of the adrenomedullin precursor from amino acids 153–185. It is believed to have opposite actions to that of adrenomedullin [112].

### 6.2. Induction of Genetic Expression

Adrenomedullin is constitutively expressed and secreted by the epithelial cells of the oral cavity. The expression is further induced when epithelial cells come in contact with microbes. Proinflammatory cytokines such as IL-1 and TNF-*α* also tend to upregulate the expression of adrenomedullin gene. Lipopolysaccharide also provides a potent stimulus to its secretion. Thus, its induction via lipopolysaccharide and cytokines proves its significant role in infection and immunity [113, 114].

### 6.3. Structure

Human adrenomedullin peptide is 52 amino acids long (Table 2) after posttransnational changes in preproadrenomedullin. It has a single disulphide bridge in between the residues at 16 and 21. Also, it has an amidated tyrosine at the carboxy terminus. Since structurally adrenomedullin peptide shares homology with calcitonin gene related peptide, it has been included into calcitonin peptide family [106].

### 6.4. Antimicrobial Action

They share some functional parallels with the *β*-defensins despite the fact that they are encoded via different genes which translate into structurally different proteins. Its salivary concentration is around 55–65 pg/mL [115]. It acts against both Gram-positive and Gram-negative bacteria of the oral cavity. The MIC of adrenomedullin is around 500 pmol/L against* P. gingivalis* while it is 12.5 *μ*g/mL against* E. coli* [115, 116]. Little is known about its antiviral activity but adrenomedullin seems to lack antifungal action [110].

### 6.5. Mechanism of Antimicrobial Action

Adrenomedullin shares its mechanism of antimicrobial activity with other cationic antimicrobial peptides. Although exact mechanism is still not elucidated, it is thought to promote intramembranous pore formation in bacterial cell membrane. With the disruption of bacterial cell membrane, there is stoppage of critical intracellular processes and finally cell death [116].

### 6.6. Other Roles

Adrenomedullin is a multifunctional peptide with a wide array of roles (Figure 2). It has potent haemodynamic effects resulting in a sustained hypotension from markedly reduced peripheral resistance [117]. It is also believed to be potent vasodilator in uterine circulation [118]. It has significant endocrine effects where it has a role in inhibiting ACTH release from pituitary, effecting secretory activity of adrenal cortex along with consequence on insulin secretion from the pancreas [119–121].

## 7. Probiotics and EAPs

Millions of microbes reside within the oral cavity and gastrointestinal track of humans. These microbes coexist with the host and are harmful to the host only when the immunity of the host is altered or there is loss of sensing and defense mechanisms of epithelial lining. These commensal bacteria are known to possess immunomodulatory capacities [122, 123]. Also, they prevent the colonization of host by pathogenic microorganisms [124]. Identification of these endogenous bacteria and their benefits has led to the development of “probiotics.” Probiotics are viable bacteria that are administered to host to competitively populate the host sites and confer health benefits. This modality is being applied for the treatment and prevention of many infectious and inflammatory diseases [125]. Such organisms include* Streptococcus salivarius*,* Lactobacillus* spp., and* Bifidobacterium* spp.; it has always been an area of speculation as to how epithelial tissues interact with commensal bacteria and/or probiotics and how they differentiate between pathogenic and nonpathogenic bacteria. Several studies have been conducted to elaborate on the interaction of probiotic organisms with epithelium. It has been suggested that there are different signaling pathways that are initiated by probiotic and pathogenic bacteria [126, 127]. The probiotic bacteria carry out immunomodulation via three ways: via an alteration in toll-like receptor (TLR) signaling, through inhibition of NF-*κ* *β* pathway, and via release of interleukin-10 cytokine [128–131]. With these mechanisms, probiotics block the proinflammatory pathways and also the production of EAPs and are protected against host defence. Thus, probiotics not only are well tolerated by host but also promote oral epithelial health, integrity, and homeostasis [132].

## 8. Future Perspective

Since the oral epithelial antimicrobial peptides are produced locally against the oral microbes and are potent in countering such insults, they are under investigations for control of oral infections. These peptides operate not only by keeping the commensal organisms in check but also by acting against pathogenic microbiota too. Thus, a systematic insight into their function, mechanism of action, and potential side effects is essential to develop them into therapeutic agents to counter oral infections such as periodontitis. The levels of these antimicrobial peptides are found to increase locally in periodontitis, and their external application may provide protection from progression of disease. Oral EAPs can be developed in a variety of ways for their therapeutic benefits. These can be produced in form of gels, mouthwashes, and gum paints for local application over periodontal tissues to not only prevent the development of periodontal disease but also reverse the existing disease. EAPs can also be developed as local drug delivery agents within the periodontal pockets to curtail the effects of periopathogenic bacteria. Because of their antiviral and antifungal effects, EAPs can be used in immunocompromised individuals against opportunistic infections such as candidiasis and herpetic gingivostomatitis. The activity of *β*-defensins against HIV holds potential for drugs that are more efficacious with lesser side effects than existing drugs for AIDS. Iseganan hydrochloride, a synthetic cathelicidin, is under investigation for prevention of ulcerative oral mucositis and has shown promising results [133]. Iseganan hydrochloride is the salt of 17-amino-acid-long synthetic protegrin-1, an 18-amino-acid antimicrobial peptide isolated from porcine leucocyte. Iseganan HCl is available as oral solution by the name of IB-367 rinse and is a broad spectrum antimicrobial and acts rapidly by disrupting cell membranes of microorganisms including bacteria, fungi, and viruses [134].

Epithelial cells are believed to behave differently when exposed to commensal and pathogenic bacteria. This could mean that antimicrobial peptide secreted from epithelial cells may have differential action against commensals and pathogens. In view of the fact that these antimicrobial peptides are broad spectrum and rapidly acting, they provide little chance of development of resistance in microbes against them. This property could be fruitful in developing novel therapeutic agents that lack resistance compared to conventional antibiotics. Finally, these antimicrobial peptides could be developed as biomarkers for oral disease diagnosis and prognosis. Therefore, there remains a whole unexplored world of therapeutic benefits of epithelial antimicrobial peptides which needs to be ventured into.

## Figures and Tables

**Table 1:** Comparison of chief characteristics of oral epithelial antimicrobial peptides.

	Human *β*-defensins			Cathelicidin (LL-37)	Calprotectin	Adrenomedullin
	hBD-1	hBD-2	hBD-3			
Genes	DEFB1 on chromosome 8	EFB4 on chromosome 8	DEFB103 on chromosome 8	CAMP gene on chromosome 3	Dimer of two peptides MRP8 and MRP14 encoded by genes S100A8 and S100A9, respectively, on chromosome 1	Adrenomedullin (ADM) gene located on chromosome 11

Number of amino acids	36–47	41	45	37	MRP8: 93 MRP14: 114	52

Secondary structure	Antiparallel *β*-pleated sheet stabilization via three intramolecular disulphide bonds formed between six cysteine amino acids			Random coil in aqueous solutions with intramolecular hydrogen bonding forms a *α*-helix secondary structure	Noncovalently complexed heterodimer of MRP8 and MRP14. Each monomer has helix-loop-helix calcium ion binding domain, EF hands, and a hinge region	Amino acid chain with a single disulphide bridge between residues 16 and 21

Expression in oral cavity	Oral mucosa, gingiva, and tongue epithelium along with salivary glands			Orogranulocytes, inflamed gingival tissues, buccal mucosa, and tongue epithelium	Oral epithelium, sulcular epithelium, and immune cells	Oral epithelium along with salivary gland epithelium and immune cells

Mechanism of antimicrobial action	Upon interaction with anion lipids of bacterial cell membrane leads to formation of multimeric pores and permeation of the membrane			Overlaps bacterial cell membrane in a carpet-like manner and dissolves it similar to detergent	Binds with trace metal ions essential for microbial functioning, thus inhibiting microbial growth	Promotes intramembranous pore formation in bacterial cell membrane

Antimicrobial spectra	Gram-negative and Gram-positive bacteria, enveloped viruses and fungi			Gram-negative and Gram-positive bacteria including many periopathogens	Gram-negative and Gram-positive bacteria including periopathogen *P. gingivalis*. Antifungal and antiviral activity	Acts against both Gram-positive and Gram-negative bacteria of oral cavity, lacking antifungal activity, antiviral activity unknown

**Table 2:** Amino acid sequences of oral epithelial antimicrobial peptides.

Human *β*-defensins	
hBD-1	DHYNCVSSGG QCLYSACPIF TKIQGTCYRG KAKCCK
hBD-2	GIGDPVTCLK SGAICHPVFC PRRYKQIGTC GLPGTKCCKK P
hBD-3	GIINTLQKYY CRVRGGRCAV LSCLPKEEQI GKCSTRGRKC CRRKK
Human cathelicidin (LL-37)	LLGDFFRKSK EKIGKEFKRI VQRIKDFLRN LVPRTES
Calprotectin	
MRP8	MLTELEKALN SIIDVYHKYS LIKGNFHAVY RDDLKKLLET ECPQYIRKKG ADVWFKELDI NTDGAVNFQE FLILVIKMGV AAHKKSHEES HKE
MRP14	MTCKMSQLER NIETIINTFH QYSVKLGHPD TLNQGEFKEL VRKDLQNFLK KENKNEKVIE HIMEDLDTNA DKQLSFFEFI MLMARLTWAS HEKMHEGDEG PGHHHKPGLG EGTP
Adrenomedullin	YRQSMNNFQG LRSFGCRFGT CTVQKLAHQI YQFTDKDKDN VAPRSKISPQ GY
